# Abrupt-onset, profound erectile dysfunction in a healthy young man after initiating over-the-counter omeprazole: a case report

**DOI:** 10.1186/s13256-021-02981-5

**Published:** 2021-07-24

**Authors:** Theodore W. Perry

**Affiliations:** North Texas VA Medical Center, Dallas, TX USA

**Keywords:** Proton pump inhibitor, Omeprazole, Erectile dysfunction, Endothelial dysfunction, Case report

## Abstract

**Background:**

Proton pump inhibitors are frequently used (and often overused) medications with adverse effects including vitamin B12 deficiency, *Clostridium difficile* colitis, and increased risk of chronic kidney disease. Erectile dysfunction is largely unrecognized as an adverse effect of proton pump inhibitors despite increasing evidence that proton pump inhibitors may contribute to impaired nitric oxide generation and endothelial dysfunction.

**Case presentation:**

A 38-year-old Caucasian man with mild hypertension and no other significant medical history developed profound erectile dysfunction within 2 days of initiating over-the-counter omeprazole therapy, with erectile function rapidly normalizing following discontinuation of the drug. At the time of the episode, the patient was on a stable dose of lisinopril and was taking no other medications or supplements. In the 2 years following the episode, the patient has had no further erectile difficulties.

**Conclusion:**

Further study of erectile dysfunction as an adverse effect of proton pump inhibitors is needed. In the meantime, proton pump inhibitors should be considered as a potential cause of erectile dysfunction in healthy young patients and as a cause or contributor to erectile dysfunction in older patients in whom erectile dysfunction is often attributed to age or comorbidities.

## Introduction/background

Proton pump inhibitors (PPIs) are indicated for the management of peptic ulcer disease and gastroesophageal reflux disease. The utility of PPIs in these settings owes to their inhibition of the hydrogen–potassium ATPase in gastric parietal cells, which decreases the release of hydrogen ions into the gastric lumen and lowers gastric acidity [1]. A number of uncommon but well-recognized adverse effects of PPIs (for example, vitamin B12 malabsorption, *Clostridium difficile* colitis, reduced bone density) are likely consequences of this primary mechanism of action [2–4]. However, the pathophysiology of other associated adverse effects (for example, increased risk of chronic kidney disease and cardiovascular events) is less clear, suggesting that PPIs likely interact with other physiologic processes [5, 6]. In light of the wide use (and often overuse) of PPIs, a more complete characterization of potential adverse effects is needed.

Erectile dysfunction (ED) most commonly affects older men (age > 40 years) and is strongly associated with risk factors including coronary artery disease, diabetes mellitus, hypertension, obesity, and smoking [7–9]. In addition, certain medications are considered frequent precipitants of ED. These medications include selective serotonin reuptake inhibitors, spironolactone, thiazide diuretics, and some H2-receptor blockers (for example, cimetidine) [10, 11]. PPIs are largely unrecognized as a potential cause or contributor to ED, despite increasing evidence suggesting impaired nitric oxide generation and endothelial dysfunction as a possible effect of PPIs [12–14]. This case report describes the abrupt onset of profound ED in a healthy young man after initiating over-the-counter omeprazole therapy, with erectile function rapidly normalizing following discontinuation of the drug.

## Case presentation

A 38-year-old Caucasian man with a history of mild hypertension and who was otherwise healthy [never smoker, body mass index (BMI) 19.6 kg/m^2^] began taking over-the-counter omeprazole 20 mg daily owing to new-onset gastroesophageal reflux symptoms. One day after initiating therapy (day 2 of therapy), the patient began to notice difficulty obtaining and maintaining an erection despite sustained libido. By day 3 of therapy, the patient was completely unable to obtain an erection. After the fourth day of therapy, the patient suspected the new medication as the culprit and discontinued use. Erectile function then returned to normal over the next several days. The patient had no history of previous ED and in the 2 years following the episode has had no further erectile difficulties. His only other medication was lisinopril 10 mg daily, for which he had been on a stable dose for 11 years. No other medications or supplements were being used.

## Discussion and conclusions

Several factors of this patient’s clinical history create a compelling argument implicating omeprazole as a potent precipitant of ED. These include the temporal relationship of the onset of ED with the initiation of omeprazole therapy, the prompt resolution of symptoms following discontinuation of the drug, the absence of other readily apparent precipitating factors, and the absence of recurrent ED in the 2 years following the episode. Limitations include the possibility of an unrecognized factor that temporally coincided with omeprazole therapy and the potential of bias (for example, anchoring) affecting the accuracy of the history. However, thorough history-taking in this case likely minimizes the impact of these limitations.

The mention of ED as a potential adverse effect of PPI use is sparse within the medical literature. Likely the best documentation of this association is a pharmacovigilance report from the Netherlands that identified 17 cases of ED between 1992 and 2015 that were suspected to be related to omeprazole use [15]_._ Of the 17 cases, 3 were in men age < 40 years with no known risk factors for ED. Two of those three men were taking omeprazole 40 mg daily, and one was taking 20 mg daily. Two of the men recovered following withdrawal of the drug, and the third man remained on the drug (with unresolved symptoms) until the end of follow-up. An earlier drug monitoring study from Sweden reported 15 cases of ED suspected to be related to omeprazole, with 2 of those cases in men age < 40 years [16].

Several mechanisms of ED related to PPI use are possible. Induction of CYP3A4 may decrease levels of testosterone in some patients; however, such a mechanism would seem unlikely to cause profound ED after 2 days of therapy. Altered function of calcium channels within the corpus cavernosum may also be suggested [17]. The most likely mechanism would seem to be endothelial vasodilatory dysfunction mediated by impaired generation of nitric oxide. Evidence suggests that PPIs inhibit the enzyme dimethylarginine dimethylaminohydrolase (DDAH), which leads to blocked degradation of asymmetrical dimethylarginine (ADMA) and consequent impaired endothelial nitric oxide generation (increased ADMA levels are considered a potential marker of endothelial dysfunction) [18]. This mechanism could have especially potent effects on erectile function in patients with genetic polymorphisms leading to reduced baseline activity of DDAH, possibly explaining the abrupt onset and profound symptoms in this patient. Such endothelial dysfunction could also largely explain the associations of PPIs with chronic kidney disease and cardiovascular events (as well as a possible association with dementia) by accounting for chronic low levels of tissue ischemia with prolonged PPI use [5, 6, 19].

In conclusion, the incidence of ED attributable to PPI use may be underrecognized. Further study is needed to better characterize the incidence of ED associated with PPI use, and whether this adverse effect is related to a mechanism of endothelial dysfunction. In the meantime, PPIs should be considered as a potential cause of ED in young and healthy patients. PPIs should also be considered as a cause or contributor to ED in older patients in whom ED is often attributed to age or comorbidities.

## Abbreviations

ADMA: Asymmetrical dimethylarginine
DDAH: Dimethylarginine dimethylaminohydrolase
ED: Erectile dysfunction
PPI: Proton pump inhibitor
